# Intraspecific relationships between floral signals and rewards with implications for plant fitness

**DOI:** 10.1093/aobpla/plab006

**Published:** 2021-01-20

**Authors:** Carla J Essenberg

**Affiliations:** Department of Biology, Bates College, Lewiston, ME, USA

**Keywords:** Communication, evolution, floral colour change, floral traits, foraging behaviour, honest signal, information, mutualism, plant reproduction, pollination, sexual dimorphism, signalling theory

## Abstract

Within-species variation in traits such as petal size or colour often provides reliable information to pollinators about the rewards offered to them by flowers. In spite of potential disadvantages of allowing pollinators to discriminate against less-rewarding flowers, examples of informative floral signals are diverse in form and widely distributed across plant taxa, apparently having evolved repeatedly in different lineages. Although hypotheses about the adaptive value of providing reward information have been proposed and tested in a few cases, a unified effort to understand the evolutionary mechanisms favouring informative floral signals has yet to emerge. This review describes the diversity of ways in which floral signals can be linked with floral rewards within plant species and discusses the constraints and selective pressures on floral signal–reward relationships. It focuses particularly on how information about floral rewards can influence pollinator behaviour and how those behavioural changes may, in turn, affect plant fitness, selecting either for providing or withholding reward information. Most of the hypotheses about the evolution of floral signal–reward relationships are, as yet, untested, and the review identifies promising research directions for addressing these considerable gaps in knowledge. The advantages and disadvantages of sharing floral reward information with pollinators likely play an important role in floral trait evolution, and opportunities abound to further our understanding of this neglected aspect of floral signalling.

## Teaser

Flowers commonly reward the animals that pollinate them with food or other benefits, but even within a plant species, not all flowers are equally generous. Curiously, in many plant species, traits such as flower size or scent differ between more- and less-rewarding flowers, enabling pollinators to discriminate against the less-rewarding flowers. Although these associations between floral signals and rewards are widespread across the plant kingdom, their fitness effects are poorly understood. This paper reviews current knowledge about relationships between floral signals and rewards and presents hypotheses to explain their evolution.

## Introduction

The signalling role of floral traits such as volatile emissions and the colour, shape and size of petals has inspired a wealth of research (e.g. Leppik 1955; Wester and Lunau 2017; Koski 2020). Biologists have considered in particular depth how these traits influence floral attractiveness to pollinators as well as pollinators’ ability to distinguish between plant species (e.g. Slaa *et al.* 2003; de Jager *et al.* 2011; Jersáková *et al.* 2012). While both of these functions are certainly important for plants’ reproductive success, additional signalling functions may play important and underappreciated roles in shaping floral traits (e.g. Lunau 2006; Leonard *et al.* 2013). In particular, floral signals frequently provide information about the relative fitness benefits, or ‘rewards’, offered by different flowers within a plant species: such signals (hereafter referred to as ‘informative floral signals’) have been found in many hundreds of species widely spread across plant taxa and appear to have evolved many times (Weiss 1995b; Delph *et al.* 1996; Dobson and Bergstrom 2000; Hansen *et al.* 2007). They may, therefore, solve common problems faced by many plant taxa, but what those problems are is uncertain.

Conversely, some common floral traits may be adaptations to withhold information about floral rewards. For example, the yellow spots found on flowers of many plant species and the close similarity in colour between pollen, anthers and sometimes petals could be adaptations to conceal information about pollen presence or absence (Lunau 2006; Xiong *et al.* 2019). Flowers also often display glossy patches that could function as nectar mimics (Lunau *et al.* 2020). However, why deception may have evolved in these cases while informative floral signals evolved in others is not known.

Although reviews are available on some specific categories of informative or deceptive floral signal, including floral signal changes over the lifetime of a flower (Weiss and Lamont 1997; Raguso and Weiss 2015), floral sexual dimorphism (Eckhart 1999; Ashman 2009), coloured nectar (Hansen *et al.* 2007), pollen scent (Dobson and Bergstrom 2000), nectar scent (Raguso 2004) and pollen mimicry (Lunau 2006), no review has unified this literature, discussing these and the other types of informative floral signal within a single framework. Consequently, no previous review has focused on the shared selective pressures associated with providing information about floral rewards. The result is that the various literatures on informative and deceptive floral signals remain largely separate, slowing the dissemination of ideas and insights about how reward information influences pollinator behaviour and pollination ecology.

Ignorance about the contexts favouring and disfavouring informative floral signals limits not only our understanding of the evolution of floral traits but also our understanding of ecological interactions within plant–pollinator networks. Informative floral signals have the potential to influence the success of species that possess them, their pollinators and other plant species with which they share pollinators. By enabling pollinators to avoid relatively unrewarding flowers within plant species, informative floral signals could raise pollinators’ foraging success. This increased foraging success, in turn, could heighten the relative attractiveness of plant species that offer informative signals, aiding them in competition with co-flowering plant species. Where common in a plant community, informative floral signals might also allow a more efficient transfer of resources from plants to pollinators because of pollinators’ reduced foraging costs. A better understanding of the factors favouring and disfavouring the evolution of informative floral signals could help predict where they are likely to occur and thus anticipate these ecological effects.

This paper seeks to integrate insights and information gained from study of all of the various types of informative floral signals documented in the literature to better understand the evolution of relationships between floral signals and rewards. I begin by highlighting the diversity of ways in which a floral signal can be associated with floral rewards and briefly summarizing the kinds of informative floral signals recorded in the literature. The next section considers the degree to which observed relationships between floral signals and rewards are the result of selection imposed by pollinators, pointing out evolutionary constraints that are likely to influence these relationships and presenting evidence of pollinator-mediated evolution.

The major, guiding question of the review, how providing floral reward information influences pollinator behaviour and, consequently, pollination success, is considered in the remaining sections. Effects on pollinator behaviour are likely to be shared between types of informative floral signals that currently are considered in largely separate literatures. Behavioural responses to reward information are therefore key to integrating these literatures. Many of the effects of those responses on plant fitness are also likely to be similar across different types of informative floral signal, although others will differ markedly depending on how the signal is connected to floral rewards. I discuss these fitness effects and the conditions favouring sharing reward information as opposed to withholding it.

The major message that emerged from my reading of this literature is that we know surprisingly little about the evolution of informative versus uninformative floral signals within plant species. The main purpose of this paper is therefore to identify hypotheses that need to be tested and questions that need to be answered.

## Literature Review

The diversity of floral signal–reward relationships and consequent breadth of the literature precluded a systematic literature review. Instead, I used a wide variety of search terms to gain entry into the literatures that have considered these traits, including those surrounding floral colour change, sexual selection and sexual dimorphism in plants, correlations between flower size and reward production and nectar- and pollen-produced cues. I used broad search terms such as ‘honest signal’ and ‘mimicry’ to locate additional papers that focused on signal–reward relationships. I focused in particular on finding information about how pollinators respond to informative floral signals and the fitness effects of providing reward information.

## Notes on Signalling Terminology

Within this paper, I shall use the term ‘floral signal’ to refer to any floral trait that pollinators can detect before probing a flower, even if that trait might have evolved for reasons other than its effects on pollinators. (I adopt this relatively broad definition because the data needed to establish floral traits’ evolutionary histories are often unavailable.) ‘Informative floral signal’ will refer to any floral trait that could allow pollinators to discriminate in favour of flowers that, on average, have superior rewards to the flowers they are discriminating against.

## Connections Between Floral Signals and Rewards

**Figure 1.**
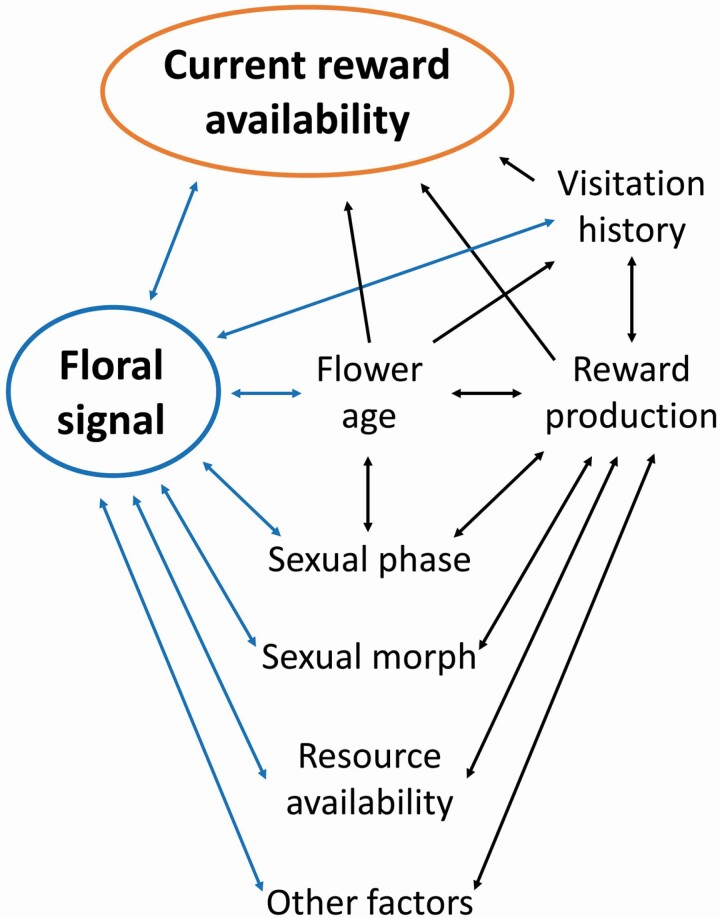
Direct and indirect causal pathways by which a floral signal can be connected with a floral reward. Each blue arrow represents a potential direct connection between the signal and another variable.

A floral signal can be associated with current floral reward availability via a variety of causal pathways (Fig. 1). In some cases, the reward itself might produce a signal, such as a distinctive scent, that pollinators can detect before probing the flower. However, in most cases the connection between current reward availability and floral signal is indirect, and the signal is associated with only one or two out of multiple variables that influence current reward availability.

**Figure 2.**
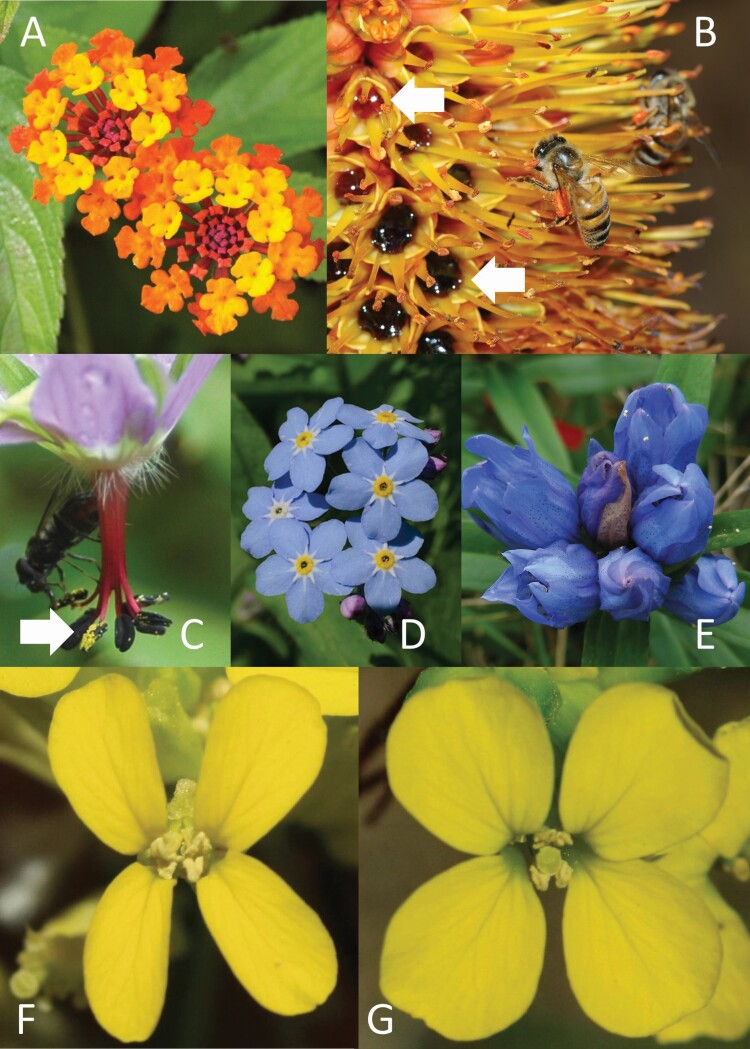
Examples of visual signals that provide or withhold floral reward information. (A) In *Lantana camara*, flowers change from yellow to red and nectar is produced only in the yellow flowers (Weiss 1995a, 1997), but some flowers do not produce nectar even in the yellow phase (Anand *et al.* 2007). (B) Phenolic compounds give the nectar of *Aloe vryheidensis* flowers a dark red-brown colour that darkens as the flowers age (nectar marked by arrows; Johnson *et al.* 2006). Photograph by Steve Johnson, previously published in Johnson *et al.* (2006), © 2006 by the Ecological Society of America, published with permission. (C) In *Geranium delavayi*, pollen (marked by arrow) contrasts in colour with the anthers and other flower parts. Photograph by Shuang-Quan Huang, previously published in Xiong *et al.* (2019), © 2019 The Authors, published with permission. (D) Anthers in *Myosotis sylvatica* are concealed inside the corolla tube, and their signalling function may have been taken over by the yellow ring of coronal scales around the mouth of the corolla tube (Lunau *et al.* 2017). This yellow ring changes from yellow to white with flower age, and availability of both pollen and nectar is greater in the yellow than in the white stage (Nuttman and Willmer 2008). Photograph by Tero Laakso, licensed for use under CC BY-SA 2.0, cropped to fit into this figure. (E) In *Gentiana triflora* var. *japonica*, degree of floral openness during the day varies with flower age, as do nectar and pollen rewards (Kato and Sakai 2008; Fusato *et al.* 2015). Photograph credit: Yusuke Fusato, Tomoyuki Itagaki and Satoki Sakai, published with permission. In *Erysimum mediohispanicum*, narrower, less rounded petals, as in (F), are associated with greater nectar and pollen production than more rounded petals, as in (G) (Gómez *et al.* 2008b; Gómez and Perfectti 2010). In addition, flowers with longer corolla tubes produce more nectar and flowers with wider corollas (i.e. longer petals) produce more pollen than smaller flowers do (Gómez *et al.* 2008a). Photographs by Francisco Perfectti, published with permission.

For example, in *Lantana camara*, flowers change colour with age, from yellow to red, and nectar is produced only in yellow flowers (Weiss 1995a, 1997; Fig. 2A). Flower colour therefore provides valuable information to pollinators. However, even yellow *L. camara* flowers vary greatly in their nectar production rates, with many producing no nectar at all (Anand *et al.* 2007).

In this section, I summarize examples documented in the literature of floral signals that are associated with floral rewards, grouping them based on how signal and reward are connected to each other. I point out some patterns in their occurrence across taxa but save discussion of their evolution and effects on pollinators and plant fitness for later sections.

### Signal is directly connected to current reward status

Floral rewards can, themselves, produce signals, potentially allowing pollinators to gain information about the current reward status of a flower. Some tropical and subtropical plant species present nectar with a conspicuous colour: although a rare trait, coloured nectar is phylogenetically widespread, being distributed across at least 13 plant orders (Hansen *et al.* 2007; Fig. 2B). Pollen is often displayed outside the flower and sometimes contrasts in colour with the anthers, suggesting that it too might be detectable through visual signals (Xiong *et al.* 2019; Fig. 2C). Less common floral rewards can also present visual signals (e.g. blue resin: Bolstad *et al.* 2010; food bodies: Dellinger *et al.* 2014).

Rewards can also produce volatiles. Pollen is often scented and, where the comparison has been made, pollen scent typically differs from the scent of the rest of the flower (e.g. Dobson and Bergstrom 2000; Flamini *et al.* 2003). Data on nectar-produced volatiles are limited but indicate that some nectar contains volatiles distinct from those of other flower parts (Raguso 2004), although the scents involved are sometimes yeast volatiles rather than volatiles produced by the plant (Herrera *et al.* 2013; Schaeffer *et al.* 2014; Yang *et al.* 2019). In species with thermogenic flowers, floral heat commonly volatilizes scent compounds and consequently is associated with high scent emissions (Lamprecht *et al.* 2002). Where heat acts as a reward for pollinators, scent can therefore serve as an informative floral signal (e.g. Seymour and Matthews 2006). Finally, in some cases, floral volatiles serve as both signal and reward. For example, male Euglossine bees collect floral volatiles to use in courtship, and many tropical orchid species rely on them for pollination, offering only perfume as a reward (Hetherington-Rauth and Ramírez 2016; Milet-Pinheiro and Gerlach 2017).

### Signal is connected to floral reward production

In many cases, intraspecific variation in floral signals is correlated with variation in reward production rather than being directly associated with the current availability of rewards (i.e. standing crop). Although these signals can provide valuable information to pollinators about floral rewards (e.g. Weiss 1995a, 1997), flowers presenting the same signals will vary in their standing crops because of different visitation histories.

Both the abundance of flower visitors and the rate at which floral rewards are made available will determine how well reward production predicts current reward availability. Reward production rates and schedules vary widely. For instance, the duration of anther dehiscence (i.e. release of pollen) varies greatly across species (e.g. Castellanos *et al.* 2006). In some species, additional mechanisms to extend the period of pollen release have evolved, including ones that release pollen in response to pollinator visits (e.g. Erbar and Leins 1995; Henning *et al.* 2018; Mione and Diaz 2020). However, pollen release can also end well before flowers close (e.g. Teixido *et al.* 2018). Nectar is commonly secreted over an extended period, and in many species, net nectar production rates increase in response to flower visits (Ordano and Ornelas 2004). However, nectar secretion rates can vary across the life of the flower, including ceasing entirely (e.g. Weiss and Lamont 1997), and flowers of many species accumulate nectar, either in the bud or overnight (e.g. Guerenstein *et al.* 2004; Antoń *et al.* 2017), making initial visits more rewarding than subsequent visits.

The connections between floral signal and reward production are often indirect: for instance, the floral signal may be associated with flower age, sexual phase or sexual morph, which in turn may be only one of several variables influencing reward production (Fig. 1).

#### Changes in floral rewards and signals with flower age and sexual phase.

Floral reward production often changes over the lifetime of a flower. In some plant species, flowers are retained for a substantial period after reward production has ceased (e.g. Weiss and Lamont 1997). Commonly, the old, unrewarding flowers are no longer receptive to pollen and have little or no viable pollen to export. The reasons why plants retain the petals of these flowers are not fully understood but could include increasing the attractiveness of the plant from a distance by increasing the size of its floral display, allowing time for pollen tubes to reach the ovary in species in which petals and style are abscised simultaneously, and/or providing protection to the developing fruit (Delph and Lively 1989; Weiss and Lamont 1997; He *et al.* 2005; Brito *et al.* 2015).

Floral rewards can also differ between sexual phases in dichogamous plants, species in which flowers go through separate male and female phases (Carlson and Harms 2006). Typically, pollen is available only during male and hermaphroditic phases, and nectar production can also differ between phases. Data available thus far suggest that male- and female-biased nectar production occur with similar frequency (Carlson and Harms 2006).

Often floral signals change with age, allowing pollinators to discriminate between the more- and less-rewarding stages (e.g. Fig. 2A, D and E; reviewed by Weiss and Lamont 1997; Raguso and Weiss 2015; Ruxton and Schaefer 2016; also see Weiss 1995b; for an exception, see Teixido *et al.* 2018). Changes with floral age in colour of all or part of the flower have been recorded in at least 38 % of angiosperm orders and 20 % of angiosperm families (Weiss and Lamont 1997). Other traits that can alter with floral age, reproductive status and reward availability include scent (reviewed by Raguso and Weiss 2015) and morphology (e.g. Fig. 2E; Eisikowitch and Rotem 1987; Jones and Cruzan 1999; Fusato *et al.* 2015). Often multiple traits change at once (e.g. Eisikowitch and Rotem 1987; Yan *et al.* 2016). The timing of these changes appears to be fixed in some plant species (e.g. Ida and Kudo 2003) while in others it can be accelerated by pollination (e.g. Zhang *et al.* 2017), nectar removal (e.g. Eisikowitch and Lazar 1987) and/or physical disturbance (e.g. being probed, even without nectar removal, Nuttman *et al.* 2006). Although dramatic changes in floral signals seem most frequently to accompany a shift to a post-sexual, unrewarding stage, they can also signal shifts between sexual phases in dichogamous species (e.g. Seymour and Schultze-Motel 1997; Carlson 2007; Davis *et al.* 2014).

#### Reward and signal differences between sexual morphs.

Floral reward availability often differs between sexual morphs and can also differ between morphs in heterostylous species (Wolfe and Barrett 1987; Eckhart 1999; Carlson and Harms 2006). In gynodioecious species, hermaphrodite plants typically offer more nectar than female plants (Eckhart 1999). In dioecious species, nectar production commonly differs between male and female plants as well, but there is no consistent trend towards one sex or the other providing more nectar. For pollen foragers, pistillate flowers are usually rewardless, although in some species they may produce inviable pollen (reviewed by Mayer and Charlesworth 1991; see also, e.g. Aranha Filho *et al.* 2009; Anderson *et al.* 2015; and papers cited within).

A variety of floral signals can differ between sexual morphs. Floral scent frequently differs between morphs, with male plants’ flowers often producing a greater quantity of volatiles and/or a different volatile composition from females’ flowers (Ashman 2009). Flower size also frequently differs between sexual morphs, with bisexual flowers and, in temperate species, staminate flowers typically having larger perianths than pistillate flowers, although pistillate flowers can also have larger perianths than staminate flowers, particularly in the tropics (Delph *et al.* 1996). Signals produced by reproductive structures, such as stamens and anthers, might also enable pollinators to discriminate between sexual morphs.

#### Other relationships between floral signals and reward production.

In many species, reward production varies across flowers even of the same age, pollination status and sexual phase (e.g. Anand *et al.* 2007). Often, variation in signals such as petal size, shape or scent is correlated with this variation in reward production, enabling pollinators to discriminate against relatively under-rewarding flowers. The most commonly documented of these informative signals is flower size: positive correlations between one or more aspects of perianth size and the volume of nectar, amount of nectar sugar and/or amount of pollen produced have been documented in many plant species and can occur across flowers within individual plants as well as among plants (e.g. Fenster *et al.* 2006; Gómez *et al.* 2008a; Benitez-Vieyra *et al.* 2010; C. J. Essenberg *et al.*, unpubl. data). In some cases, both flower size and either flower shape or scent are related to reward production (Møller 1995; Gómez *et al.* 2008a; Gómez and Perfectti 2010; Knauer and Schiestl 2015; Gervasi and Schiestl 2017). Occasionally, flower colour polymorphisms are also associated with reward variation (Carlson and Holsinger 2013; Varga and Soulsbury 2017).

## When Is Providing or Withholding Information an Adaptation?

A challenge to interpreting relationships between floral signals and rewards is that in many cases we do not know whether they represent adaptations, either to communicate or to deceive. In this section, I consider constraints on the evolution of floral signal–reward relationships and evidence for pollinator-mediated evolution of those relationships.

### Evolutionary constraints on relationships between floral signals and rewards

#### Developmental and genetic constraints.

Relationships between signal and reward production across flowers within an individual plant require either that there are regulatory pathways that influence both traits and differ in expression across flowers or that both traits independently respond in the same way to variation in the floral environment (e.g. local availability of water, light or photosynthate). Across plants within a population, genetic variation can also account for variation in floral traits, including floral signals and rewards (Gómez *et al.* 2009; Parachnowitsch *et al.* 2019). Genes with pleiotropic effects on both floral signals and rewards and genetic linkage between genes influencing these traits could generate signal–reward correlations across plants within a population even if those relationships were not adaptive. However, in the absence of pleiotropy or genetic linkage, sexual recombination could prevent associations between alleles for floral rewards and floral signals from arising in a population.

The availability of consistent changes in the regulatory environment across the lifetime of a flower might explain why concerted changes in reward production and floral signals have evolved many times (Raguso and Weiss 2015). Similarly, differences in gene expression across flowers of different sexes may explain why intersexual differences in both reward production and floral signals occur in many species.

Research on floral development and genetics has also identified several examples of pleiotropy or genetic linkage connecting signal and reward traits. For example, genes influencing flower size occur at or near the same locations as genes controlling sexual morph in at least some species (Krizek and Anderson 2013). In several plant taxa, single quantitative trait loci influence both petal size or shape and nectar traits such as volume or concentration (Smith 2016). In addition, some regulatory pathways simultaneously govern the growth of multiple flower parts and therefore might generate positive correlations between sizes of petals, anthers and nectar-producing tissues within a plant.

#### Selection on other functions.

Structures, pigments and volatiles serving as signals sometimes serve other functions as well, and selection on those functions may constrain their evolution. In addition to presenting signals to pollinators, flower petals and sepals provide protection for reproductive structures. This function can select for differences between petals of different floral sexual morphs and changes in petal morphology over a flower’s lifetime (e.g. Delph *et al.* 1996; He *et al.* 2005). For example, the need to accommodate a large gynoecium may explain most cases in which pistillate flowers have larger petals than staminate flowers do: out of 53 plant taxa, Delph *et al.* (1996) found that almost all species with larger perianths in pistillate than in staminate flowers also had larger gynoecia than androecia. (Species with larger perianths in staminate than in pistillate flowers usually had larger androecia than gynoecia, although there were many exceptions.)

**Figure 3.**
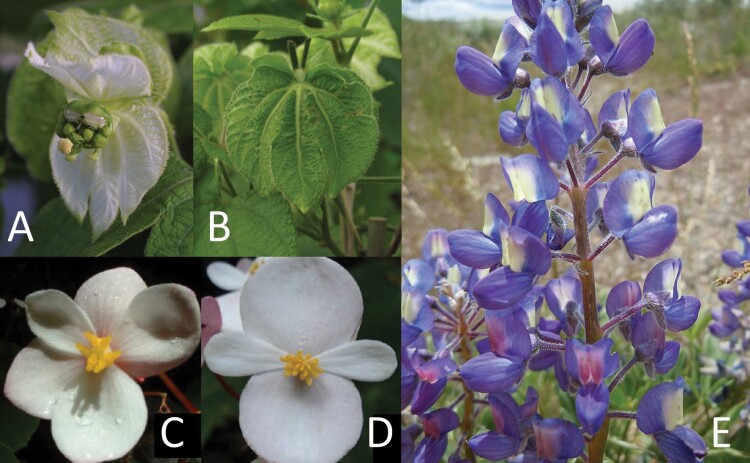
Relationships between floral signals and rewards may or may not be adaptations to provide or withhold information from pollinators. The change from white (A) to green (B) bracts in *Dalechampia scandens* inflorescences appears to benefit the plant by raising photosynthetic rate during seed maturation, although it may help pollinators avoid old inflorescences as a by-product (Pélabon *et al.* 2015). Photographs previously published in Pélabon *et al.* (2015), © 2015 The Authors, published with permission. In *Begonia fernandocostae*, the close resemblance between the gynoecium of (C) rewardless, pistillate flowers and the androecium of (D) rewarding, staminate flowers could be an adaptation to encourage ‘mistake’ visits to pistillate flowers. Photographs previously published in Wyatt and Sazima (2011), licensed for use under CC BY 3.0, modified to alter panel labels. (E) The change from a yellow to purple banner petal spot in *Lupinus argenteus* flowers, which corresponds with a reduction in reward availability, appears to benefit plants by causing pollinators to visit more flowers before leaving (Gori 1989). Photograph by Matt Lavin, licensed for use under CC BY-SA 2.0.

Floral pigments can have a variety of functions other than signalling (e.g. Fig. 3A and B). Pigments expressed in petals can aid in defending plants against herbivores (Strauss and Whittall 2006). Similarly, the pigments giving *Aloe vryheidensis* nectar its dark-brown colour (Fig. 2B), although they increase the flowers’ attractiveness to their bird pollinators, may function primarily to discourage nectar thieves by making the nectar unpalatable to them (Johnson *et al.* 2006). The characteristic yellow colour of pollen is, in many species, a consequence of yellow flavonoid pigments possessed by all angiosperm pollen, which provide protection from ultraviolet radiation (Lunau 2006).

#### Selection imposed by eavesdroppers.

The same floral traits that direct pollinators to particularly rewarding flowers can also influence the behaviour of herbivores and pollen and nectar thieves (Irwin *et al.* 2004; McCall and Irwin 2006; Strauss and Whittall 2006). For example, having larger corollas or other signal structures (e.g. showy bracts), while often increasing pollinator visitation rates, can also increase attractiveness to florivores and seed predators (e.g. McCall and Barr 2012; Perez-Barrales *et al.* 2013). Selection from antagonists could therefore counter advantages obtained by increasing flowers’ salience to pollinators, perhaps favouring uniformity in floral signals. In addition, selective pressure from pollen thieves could favour cryptic pollen and anthers (Xiong *et al.* 2019). Conversely, variation in the costs of being detected by antagonists might favour variation in salience of floral signals in some cases. For example, a monoecious plant might benefit from making pistillate flowers less salient than staminate flowers to pre-dispersal seed predators.

### Evidence of pollinator-mediated evolution of signal–reward relationships

Data providing insight into the evolutionary origins of signal–reward relationships are scarce, but a handful of cases suggest that pollinator responses can shape these relationships. In some cases, the floral traits themselves provide persuasive evidence of pollinator-mediated evolution. For example, in several plant taxa (e.g. *Begonia*; Fig. 3C and D), gynoecia of rewardless pistillate flowers closely resemble different parts of the rewarding staminate flowers (such as the androecium or petals; Bawa 1980; Schemske and Agren 1995; Ghosh and Pal 2017; Krishna and Somanathan 2018). Although only circumstantial evidence, the close correspondence between different structures in the two morphs suggests that they have evolved to deceive pollinators.

A few comparative studies have also found patterns consistent with pollinator-mediated evolution of signal–reward relationships (e.g. Delph *et al.* 1996; Okamoto *et al.* 2013; Benitez-Vieyra *et al.* 2014; Ohashi *et al.* 2015; Hossaert-McKey *et al.* 2016). For example, Ohashi *et al.*’s (2015) survey of 219 Japanese plant species found that, after controlling for phylogenetic relationships, flowers of species that received abundant bee visits underwent larger changes in colour as visible by bees than species pollinated by other taxa. These results suggest that floral colour change may often evolve as a signal to bee pollinators. Conversely, Hossaert-McKey *et al.* (2016) found evidence for selection in favour of deception in a comparative study of seven dioecious fig species. Species in which the rewarding male and rewardless female plants bloomed at the same time had indistinguishable floral volatile compositions, whereas in species with asynchronously blooming male and female plants, the floral volatile compositions differed between sexes.

## Pollinator Responses to Floral Reward Information and Implications for the Evolution of Signal–Reward Relationships

This section focuses on ways in which informative floral signals influence pollinator behaviour and the resulting selective pressures favouring either providing or withholding floral reward information. Pollinators presumably neither know nor care whether the floral signals they use to identify rewarding flowers and plants are associated with sexual morph, flower age or any other trait aside from the presence of the resource they are seeking. Investigations of pollinators’ behavioural responses to informative floral signals could therefore be an area of common ground for researchers endeavouring to understand how these traits evolve.

The following sections are organized around five different aspects of pollinators’ foraging behaviour that informative floral signals could influence: (1) their choice of flowers to visit, (2) the number of flowers they visit before leaving a plant, (3) which plants they visit, (4) how likely they are to switch between plant species and (5) how often they switch between sexes, in plant species that have separate sexual morphs. Some sections focus on floral signals that provide within-plant information, while other sections consider between-plant information. Most sections begin with a discussion of the signals’ effects on pollinator behaviour and then discuss the selective pressures that these pollinator responses impose on plants.

I have worded the text in terms of how informative floral signals influence pollinator behaviour and plant fitness. However, the ideas can be turned around to consider how withholding information about floral rewards would influence pollinator behaviour and plant fitness: i.e. if providing information about floral rewards reduces plant fitness, that should select for withholding information and vice versa.

## Effects of informative signals on flower choice

### Behavioural data on informative floral signals’ effects on flower choice

Informative floral signals may enable pollinators to raise their fitness by discriminating in favour of flowers that offer them greater fitness benefits against flowers that have less to offer. While it may be tempting, therefore, to assume that any signal difference between more- and less-rewarding flowers will increase visitation rates to the more-rewarding types and reduce visitation to the less-rewarding types, the reality is somewhat more complex.

Much of this complexity stems from the existence of multiple, sometimes redundant sources of information about floral rewards (for reviews considering floral signal complexity, see Leonard *et al.* 2012; Junker and Parachnowitsch 2015). As described earlier, the signals of more- and less-rewarding categories of flowers, such as flowers of different age or flowers of different sexual morph, often differ in multiple ways. In addition, pollinators can use cues that are outside of plants’ control to discriminate between more- and less-rewarding flowers. These include volatiles released by nectar yeasts (Herrera *et al.* 2013; Schaeffer *et al.* 2014; Yang *et al.* 2019), scents left on flowers by recent flower visitors (e.g. Giurfa *et al.* 1994; Yokoi and Fujisaki 2007; Goulson 2009; Pearce *et al.* 2017), heightened humidity and carbon dioxide concentrations in the headspace of recently opened flowers (Goyret *et al.* 2008; von Arx *et al.* 2012) and even changes in floral electrical potential caused by recent visits (Clarke *et al.* 2013).

When multiple cues are associated with floral rewards, an individual signal may have little effect on pollinators’ behaviour. For example, in *Tibouchina sellowiana* and *T. pulchra*, flowers change both scent and colour (from white to pink) between their first and second days. Placing second-day petals or scent on first-day flowers or vice versa had only a small effect on the flower choices of pollinating bees, which continued to prefer day one flowers regardless of which petals or scent they displayed (Pereira *et al.* 2011). Presumably the pollinators were attending to additional cues that the researchers were not able to manipulate.

Conversely, pollinators can fail to discriminate between more- and less-rewarding flowers in spite of signal differences between them. For example, bumblebees that had foraged on *Mimulus guttatus*, which offers pollen as its only reward, preferred the scent of fertile anthers over the scent of sterile anthers. However, when presented with whole flower scent, they failed to discriminate between male-fertile and male-sterile flowers (Carr *et al.* 2015; Haber *et al.* 2019). Instead, both naïve and experienced bumblebees exhibited strong preferences for the scent of flowers from outbred plants. On average, outbred plants provided better pollen rewards, so the scent difference between inbred and outbred plants was also an informative floral signal, but it was considerably less reliable than the signals that the bees ignored.

A possible explanation for these results is that the cost of visiting the relatively infrequent sterile flowers on outbred plants was too small for it to be worth the extra time required to discriminate against them. Bees are subject to speed-accuracy trade-offs when discriminating between similar cues, and they do sometimes sacrifice accuracy in favour of speed when a perceptual task is difficult and the cost of making an error is low (Chittka *et al.* 2003; Dyer and Chittka 2004). The volatiles that pollinators could have used to discriminate between flowers with fertile versus sterile anthers were only small components of the total floral scent in *M. guttatus* (Haber *et al.* 2019). In contrast, flowers of outbred plants released large quantities of the volatile β-*trans*-bergamotene, which bees used to distinguish them from inbred flowers.

In general, reward-produced signals are also likely to constitute minor components of floral signals, and pollinators may, therefore, be particularly likely to ignore them. Experimental manipulations of nectar and pollen presence have nonetheless demonstrated that flower visitors sometimes detect the presence of these rewards, although in other cases they do not (e.g. Ashman *et al.* 2000; Goulson *et al.* 2001; Hansen *et al.* 2006; Johnson *et al.* 2006; Howell and Alarcon 2007; Bolstad *et al.* 2010; Zhang *et al.* 2012; Raihan and Kawakubo 2014; Brunet *et al.* 2015).

Other informative floral signals often have substantial effects on pollinators’ flower choice. Flower manipulation experiments on *Fragaria virginiana* found that pollinators (mostly small, generalist bees) used multiple signals to discriminate between the more-rewarding bisexual flowers and less-rewarding pistillate flowers, including petal length, petal width and the presence of anthers (Ashman 2000; Ashman *et al.* 2000; Ashman *et al.* 2005). Similar experiments have tested pollinator preferences for flower size and/or shape in several other plant species. In nearly all cases, experienced pollinators exhibited preferences for the size and/or shape that was associated with greater rewards (Møller 1995; Armbruster *et al.* 2005; Fenster *et al.* 2006; Gómez *et al.* 2008b; see also Keasar *et al.* 2006).

### Fitness effects of focusing pollinators’ services on a subset of a plant’s flowers

**Figure 4.**
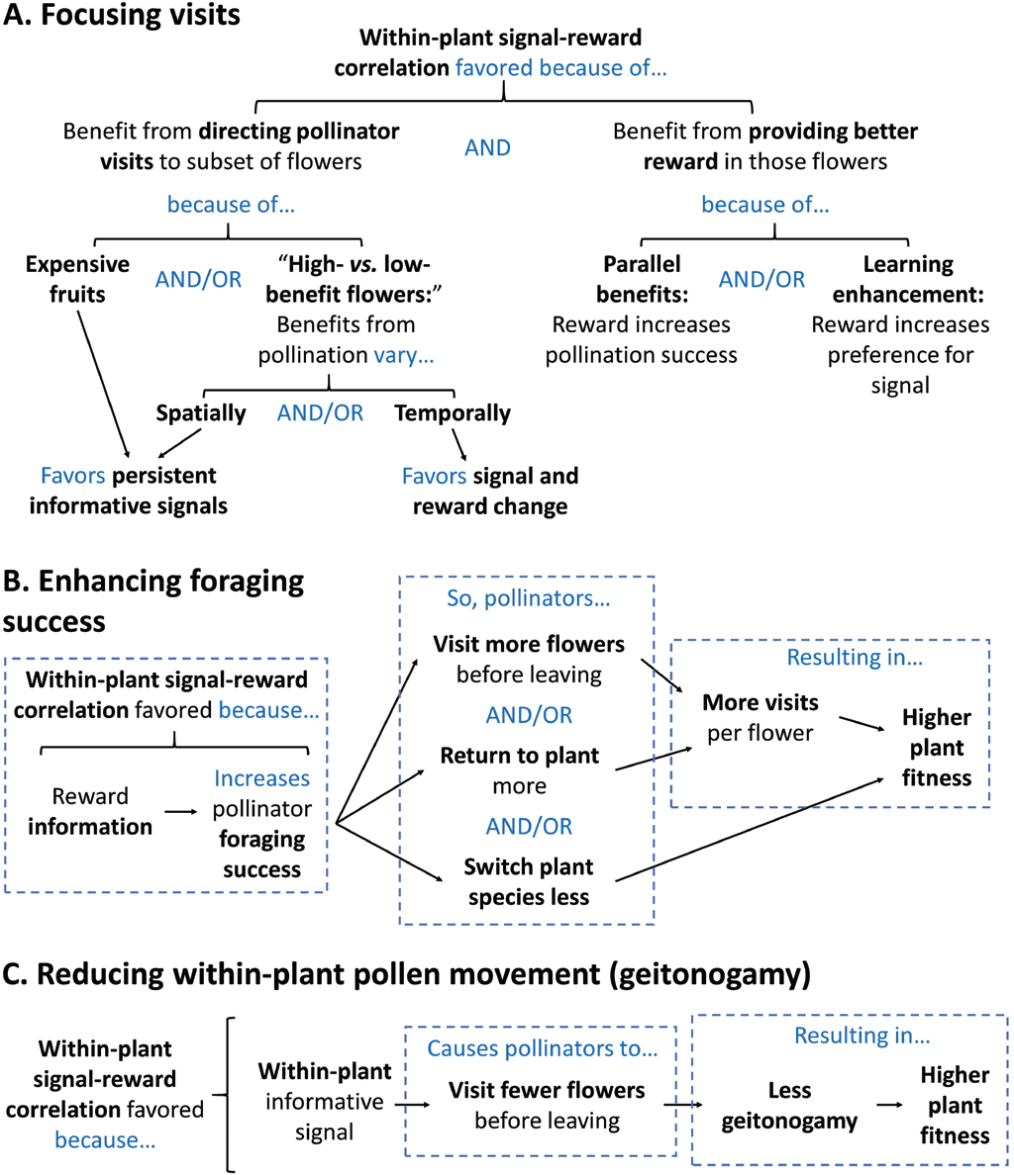
Hypothesized mechanisms favouring floral signals that provide within-plant reward information to pollinators. Conditions opposite those shown may favour uninformative floral signals. The flowcharts should be read from top to bottom (A) or left to right (B and C). Sections enclosed in dashed lines should also be read top to bottom. Arrows, brackets and text in blue indicate connections between ideas and variables. Key ideas are emphasized using bold font. Note that changes in the number of flowers a pollinator visits before leaving the plant can have opposing effects on plant fitness, which appear in (B) and (C). The effect of informative signals on this behaviour is variable and poorly understood.

Many informative floral signals provide information about within-plant variation in floral rewards. Assuming that pollinators attend to those signals, one of their effects should be to increase the number of pollinator visits that go to the more-rewarding flowers on a plant at the expense of its less-rewarding flowers. Indeed, as described in the previous section, more-rewarding flowers often do receive more flower visits than nearby flowers with poorer rewards. In this section, I consider the fitness consequences to a plant of directing pollinator visits to particular flowers (summarized in Fig. 4A). I ignore, for the moment, how informative floral signals might influence the attractiveness of the plant as a whole and the number of flower visits a pollinator makes before moving to another plant, both of which are considered in subsequent sections.

#### Why not use floral signals alone?.

Before discussing why plants might benefit from directing pollinator visits to a subset of their flowers, I will suggest two reasons why they might provide better rewards in those flowers in addition to more salient signals rather than relying on signal differences alone.

The first reason I will call “parallel benefits” (Fig. 4A). Just as increasing the attractiveness of a flower’s signals can increase pollen import and export by increasing the number of visits it receives, increasing the quantity of floral rewards a flower produces can have the same effect by increasing the quality of each visit. Pollinators often spend more time handling flowers that are more rewarding, compared to less rewarding flowers, and this can translate into greater pollen transfer per visit (e.g. Jersáková and Johnson 2006; Jersáková *et al.* 2008; Brandenburg *et al.* 2013, although see Misaki *et al.* 2018). Therefore, if some of a plant’s flowers have the capacity to deliver higher fitness benefits from pollen delivery and/or export than other flowers, then a plant’s best strategy might be to offer both more attractive advertisements and more rewards in those ‘high-benefit’ flowers compared to ‘low-benefit’ flowers.

The second reason is “learning enhancement” (Fig. 4A). Even if floral rewards do not influence the quality of individual flower visits, offering better rewards in ‘high-benefit’ than in ‘low-benefit’ flowers in addition to more attractive signals could increase a plant’s fitness by strengthening pollinators’ preferences for the signals presented by ‘high-benefit’ flowers. Pollinators can often adjust their signal preferences rapidly in response to rewards. For example, in an experiment in which naïve pipevine swallowtail butterflies visited yellow and red *L. camara* flowers, Weiss (1997) found substantial effects of nectar rewards on their flower choices within the first 10 flower visits they made. If the young, yellow flowers contained nectar and old, red flowers did not, the butterflies visited a significantly greater proportion of yellow flowers than if yellow flowers did not provide nectar.

#### Why direct pollinators to a subset of flowers?.

Some flowers on a plant may provide greater benefits from pollinator visits than others; in other words, there may be “high- vs. low-benefit flowers.” For example, flowers that have not yet been visited or have received few visits will often benefit more from pollinator visits than flowers that have received more visits. The number of ovules fertilized and amount of pollen exported per visit typically decline with successive pollinator visits to a flower, making the first few visits most valuable to the plant (e.g. Aizen and Basilio 1998; Hiei and Suzuki 2001; Morris *et al.* 2010; Vidal *et al.* 2010). Beyond a certain number of flower visits, fitness (especially female fitness) may even be reduced by further visits because of pollinator-caused damage or pollinator-vectored pathogens (e.g. Morris *et al.* 2010; McArt *et al.* 2014; Sáez *et al.* 2014; Russell *et al.* 2019).

Ceasing reward production and changing floral signals in response to flower visits or pollination could be an effective strategy for concentrating flower visits on unvisited flowers. As mentioned earlier, in some plant species, signal changes and reductions in reward production occur in response to pollination or pollinator visits, although many hours may be required to complete the change in floral traits (Eisikowitch and Lazar 1987; Nuttman and Willmer 2003; Nuttman *et al.* 2006; Carlson 2007; Zhang *et al.* 2017). Signals produced by depletable rewards (such as nectar or pollen) could achieve a similar purpose but without the time lag, if rewards were produced (or, in the case of pollen, released from anthers) only early in the flowers’ life. Even changes in signals and rewards that happen at a fixed flower age can increase the proportion of visits made to unvisited flowers, because young flowers have had less opportunity to receive flower visits. In fact, in plants with floral signal changes, the post-change flowers do typically have much less pollen available to export and are much more likely to have received pollen on their stigmas than pre-change flowers (e.g. Sun *et al.* 2005; Pereira *et al.* 2011). Stigma receptivity and pollen viability can also be lower in post-change flowers (e.g. Casper and La Pine 1984; Niesenbaum *et al.* 1999; Lippi *et al.* 2011).

In dichogamous species, changes in floral signals over a flower’s lifetime could be explained by differences in the reliance of male versus female components of plants’ fitness on pollinator visits. For example, in the protandrous herb *Chrysothemis friedrichsthaliana*, in which male-phase flowers produce more nectar than female-phase flowers and have a different colour, the number of seeds fathered by a flower increased with the number of simulated hummingbird visits it received (from 1 to 3 visits), whereas female fecundity did not (Carlson 2007). Many researchers have suggested that male reproductive success should, in general, rely more on pollinator visits than does female reproductive success (Bell 1985; Delph *et al.* 1996; Carlson 2007), which might help to explain why staminate flowers tend to be larger and produce greater volatile emissions than pistillate flowers, at least in temperate plants (Delph *et al.* 1996; Eckhart 1999; Ashman 2009). Although that idea remains open to debate, studies that have measured selection on floral traits through male versus female function have often found intersexual differences in selective pressures (Ashman and Morgan 2004; Delph and Ashman 2006; Karron and Mitchell 2012).

In some plant species, pollinators remain on flowers or inflorescences while they transition from one sexual phase to another. If the female phase came first, then the plant could benefit from signals and rewards that preferentially attracted pollinators to the female phase and encouraged them to leave during the male phase. This strategy is, in fact, seen in monoecious figs, in which inflorescences are attractive and offer egg-laying sites in their female phase only. The progeny of their pollinators emerge during the male phase and depart to find female-phase inflorescences for their own eggs (Kjellberg *et al.* 2001). (For another example, see the beetle-pollinated plant *Victoria amazonica*; Seymour and Schultze-Motel 1997).

Signal differences between staminate and pistillate flowers may reflect differences in the reliance of male versus female components of fitness on pollinator visits. However, in species with bisexual flowers, informative floral signals that cause pollinators to discriminate against certain flowers across their entire lifetime are more difficult to explain. One possibility is that local availability of resources might vary across flowers within plants, for instance because of differences in proximity to productive leaves. If this were the case, pollination services to better-resourced flowers might be more valuable to plants than pollination of more poorly resourced flowers. The fitness cost of investments in signals and rewards, in the form of reduced capacity to provision seeds and fruit, could also be lower in more highly resourced flowers, further strengthening selection in favour of informative floral signals. However, I am not aware of studies that have tested this idea.

In each of the cases described so far, the flowers vary in the benefits they can provide to the plant. However, plants with expensive fruits might benefit from concentrating pollinator visits on a subset of flowers even if all flowers were identical. My reasoning for suggesting this hypothesis is that the cost per seed dispersed could be reduced substantially by producing many seeds in each fruit. As a result, a plant might benefit from producing fruits only from flowers that had received many pollinator visits. In times of pollinator scarcity, species with expensive fruits might, therefore, benefit from concentrating pollinator visits on a subset of flowers, allowing them to package their seeds into a smaller number of fruits. In times of pollinator abundance, even the least rewarding, least attractive flowers might receive enough flower visits to set fruit. However, I am not aware of any research testing this hypothesis.

#### Some promising directions for future research.

Few of the hypotheses I present about fitness effects of informative floral signals have been adequately tested even in a single species, so opportunities to increase our understanding of these traits are plentiful. Furthermore, some of the needed data could be obtained relatively easily. Manipulation of floral signals and rewards could test whether plants actually have ‘high-benefit’ versus ‘low-benefit’ flowers (i.e. flowers that benefit more (or less) from both signal attractiveness and floral rewards) and, if so, whether informative floral signals direct pollination services to the ‘high-benefit’ flowers. Male fitness is difficult to assess, particularly when you want to compare flowers that share the same genotype, but measuring a proxy such as number of pollen grains removed and number of dye particles transferred to other flowers would provide some insights. Simulated pollinator visits, such as those carried out by Carlson (2007), who inserted hummingbird beaks into flowers, could provide information about how visit number influences fitness of more- versus less-attractive types of flower and has the advantage that it allows assessment of both male and female fitness.

Simulated pollinator visits could be particularly helpful for determining whether a plant’s total reproductive output is maximized by focusing visits on a subset of flowers or spreading the same number of visits evenly across flowers. The ‘expensive fruits’ hypothesis predicts that focusing visits should be beneficial for plants in which informative floral signals distinguish flowers of the same age and sex, whereas flowers without informative floral signals or with reward-produced signals (and similar reward production across flowers of the same age) should benefit from a more even distribution of visits across flowers. The ‘expensive fruits’ hypothesis also predicts that persistent informative signals (as opposed to signals that change over the flower’s lifetime) should be more common in species with more expensive fruits, a prediction that could be tested in a comparative study.

## Effects of informative signals on the number of flowers visited per plant visit

### Behavioural data on effects of signals that provide information about within-plant variation in floral rewards

Only a few studies have measured the effects of informative floral signals on the number of flower visits per plant visit, and their results vary. Two laboratory experiments with artificial flowers and plants found that informative signals reduce the number of flower visits per plant visit. Kudo *et al.* (2007) trained bumblebees to associate yellow flowers with sugar rewards and red flowers with the absence of rewards. The bumblebees then foraged on artificial plants. When both unrewarding and rewarding flowers were presented in the same artificial inflorescences, informative signals (i.e. yellow = rewarding, red = empty) reduced the number of flower visits per plant visit. However, when entire inflorescences were either rewarding of unrewarding, the flower colours had no significant effect on the number of flower visits per plant visit. In a similar experiment, Essenberg *et al.* (2019) found that bumblebees trained to expect rewards in large but not small flowers made fewer flower visits per plant visit if unrewarding flowers were small than if they were the same size as rewarding flowers.

In species that retain flowers in an unrewarding, post-sexual phase, the number of visits to young, rewarding flowers may be more relevant for plant fitness than the total number of flower visits. Kudo *et al.* (2007) found no significant difference in number of visits to rewarding flowers between plants with and without informative signals. Two additional experiments, one using artificial flowers and one using *Lupinus argenteus* plants in the field, found that pollinators visited more rewarding flowers per plant visit if the plant presented informative signals than if rewarding and unrewarding flowers presented the same signals (Gori 1983; Makino and Ohashi 2017).

### Fitness effects of changes in the number of flower visits per plant visit

A change in the number of flowers a pollinator visits per visit to a plant can either increase or reduce the plant’s fitness (as shown in Fig. 4B vs. 4C). If pollinators were scarce, an increase in the number of flowers each pollinator visited before leaving the plant could raise the plant’s reproductive success by increasing the per-flower visitation rate. Gori (1989) found just that effect in *L. argenteus* (Fig. 3E): when pollinators were scarce, the increased number of visits to pre-change flowers per plant visit in plants exhibiting floral colour change was associated with higher reproductive success in those plants compared to ones modified to represent a strategy without colour change.

However, an increase in the number of flower visits per plant visit can also increase the plant’s rate of geitonogamy, or pollination by other flowers within the same plant, which can have substantial negative effects on plant fitness (de Jong *et al.* 1993; Angeloni *et al.* 2011). Those effects are much more severe in some plant species than others. For instance, a fully self-compatible species in a population with a low rate of inbreeding depression might suffer very little harm from geitonogamy. At the other end of the spectrum, in some self-incompatible plant species self-pollen not only fails to pollinate flowers but can actually prevent those flowers from being pollinated by compatible pollen, either because the self-pollen clogs the stigma or because the stigma ceases to be receptive in response to receiving pollen, even if that pollen is incompatible (de Jong *et al.* 1993).

### Directions for future research

Given how few data are available on how informative floral signals influence the number of flower visits per plant visit, the most urgent need is to explore that relationship. Kudo *et al.* (2007) identified one variable that influenced pollinators’ responses to informative floral signals: whether rewarding and unrewarding flowers are in separate inflorescences or not. Inflorescence architecture as well as the distribution of rewarding and unrewarding flowers within inflorescences are also likely to matter, because they can influence the order in which pollinators encounter each type of flower as well as their ability to learn the locations of unrewarding flowers (see Harder *et al.* 2004).

## Effects of informative signals on plant choice

### Behavioural data on effects of informative floral signals on attractiveness of a floral display

Informative floral signals may or may not influence the attractiveness of entire plants to pollinators. Floral signals that distinguish more- from less-rewarding morphs in dioecious, gynodioecious or other polymorphic species can influence visitation rates at the level of the entire plant simply by making all of a plant’s individual flowers relatively attractive (or unattractive) to pollinators. However, informative floral signals that vary within individual plants, such as signals that change over the lifetime of each flower, might influence pollinator’s flower choices without influencing which plants they approach.

For example, studies of several plant species with floral colour change accompanying reductions in floral reward production have found that plant visitation rates are related to flower number but not to the proportion of post-change flowers (Gori 1989; Oberrath and Bohning-Gaese 1999; Kudo *et al.* 2007; see also Ollerton *et al.* 2007; Fusato *et al.* 2015). Additional evidence that pollinators can ignore informative floral signals when selecting plants comes from Kudo and colleagues’ (2007) laboratory experiment simulating floral colour change. Adding a ring of red flowers to an artificial inflorescence was as effective at boosting the inflorescence’s attractiveness to bumblebees as adding a ring of yellow flowers even though the bumblebees had been trained to expect rewards in yellow but not red flowers.

However, pollinators apparently do use flower colour when selecting between patches of flowers in some situations. In the dichogamous herb *Saponaria officinalis*, pollinators were more likely to make their first visit to patches of the white male-phase flowers than to patches of the less-rewarding, pink, female-phase flowers, whereas they did not discriminate against patches of the much paler female-phase flowers from plants grown in the shade (Davis *et al.* 2014).

Pollinators can also use average flower size to inform their plant choices. Essenberg *et al.* (2019) found that bumblebees trained to expect rewards in large flowers but not small ones made fewer visits to artificial plants with two large, rewarding and two small, unrewarding flowers than to deceptive plants with four large flowers, two rewarding and two unrewarding. Similarly, field experiments in *Raphanus raphanistrum* and in two *Delphinium* species found that artificially reducing the size of a plant’s flowers reduced pollinators’ approach rate (Conner and Rush 1996; Ishii and Harder 2006).

Many additional studies have found that, in populations with floral traits distinguishing more- from less-rewarding flowers, plants with a greater proportion of the more-rewarding flowers experience higher visitation rates (Casper and La Pine 1984; Cruzan *et al.* 1988; Delph and Lively 1992; Eckhart 1992; Corff *et al.* 1998; Ida and Kudo 2003; Sun *et al.* 2005). However, in these studies, floral signals and rewards are confounded, making it difficult to assess the effect of the floral signal. For example, a plant with many young, rewarding flowers might receive more visits from pollinators than one with mainly old, unrewarding flowers simply because pollinators remember the first plant’s location as a place where they found abundant floral rewards and return to that location frequently. In that case, attributing the difference in visitation rates received by the two plants to a difference in their floral signals would be a mistake.

### Effects of informative floral signals on the rate of return visits to plants

Signals that direct pollinators to particularly rewarding flowers within a plant presumably increase the pollinators’ foraging success. This increased foraging success could cause pollinators to return to plants offering informative floral signals more frequently than to plants withholding reward information (Fig. 4B). For example, pollinators sometimes remember locations of particularly rewarding plants and return to those locations more frequently than to less-rewarding plants (Cartar 2004; Makino and Sakai 2007).

To my knowledge, only one study has tested this hypothesis. In a laboratory experiment, Makino and Ohashi (2017) found that over successive foraging bouts, bumblebees decreased their visitation rate to artificial plants in which rewarding and unrewarding flowers offered identical cues and increased their visitation rate to plants in which unrewarding flowers differed in colour from rewarding flowers. Exchanging locations of these two plant types showed that bees had learned to prefer the locations of the plants with informative floral signals, rather than their combination of flower colours.

### Conditions required for evolution of informative floral signals that allow pollinators to discriminate against relatively unrewarding plants

Floral traits that allow pollinators to distinguish between more- and less-rewarding plants should benefit the more-rewarding plants by increasing the number of pollinator visits they receive. However, that benefit comes at the cost of less-rewarding members of the population. As a result, alleles that allow less-rewarding plants to mimic the floral signals of more-rewarding plants should be favoured because of the reproductive advantages they provide to individuals with relatively poor rewards. This selective pressure should erode developmental and genetic linkages between floral signals and rewards in favour of alleles that enable plants to produce highly attractive signals regardless of their floral rewards.

**Figure 5.**
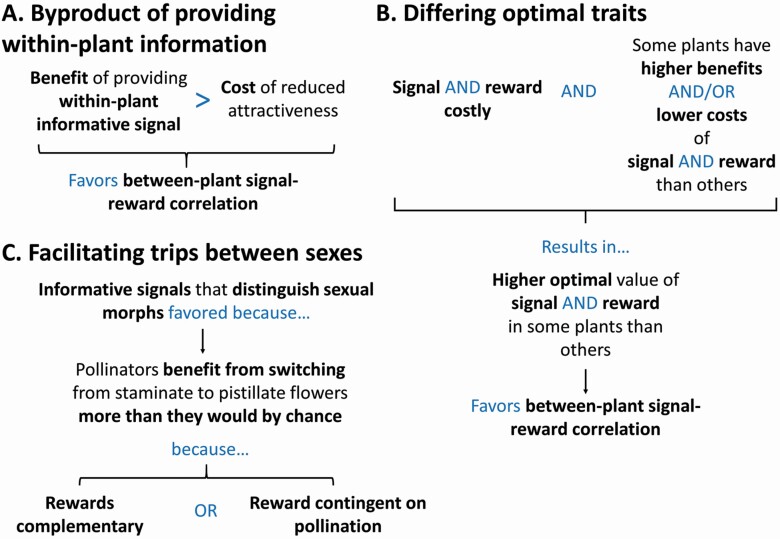
Hypothesized mechanisms favouring informative floral signals that allow pollinators to discriminate against relatively under-rewarding plants (A, B, and C). Conditions different from those shown may favour mimicry of more-rewarding plants by less-rewarding plants. The flowcharts should be read from top to bottom. Arrows, brackets and text in blue indicate connections between ideas and variables. Key ideas are emphasized using bold font.

What, then, could account for signal differences between more- and less-rewarding plants? For informative floral signals that provide within-plant information, benefits of providing that information might compensate for the costs, to less-rewarding plants, of presenting a relatively unattractive floral display (Fig. 5A). However, some other explanation is needed for informative floral traits that solely distinguish more- from less-rewarding plants.

That explanation could be higher fitness costs and/or lower fitness benefits of presenting attractive signals in less-rewarding plants in comparison with more-rewarding plants (Broom *et al.* 2013; Zollman *et al.* 2013; Sun *et al.* 2018; see also Cohen and Shmida 1993). Provided that making signals attractive was sufficiently costly, the optimal signal presented by less-rewarding plants could be less attractive than the optimal signal presented by more-rewarding plants (Fig. 5B).

To consider how differences in signal costs or benefits between more- and less-rewarding plants could come about, we have to consider selection on both floral rewards and floral signals, because plants can alter both. To favour an informative signal, the same plants in a population need to experience higher costs and/or lower benefits of both increasing floral signal attractiveness and increasing floral rewards.

Fitness costs of investments in signals and rewards could be correlated with each other because of overlap in the resources required for each. For example, both increasing petal size and increasing nectar rewards require an increased investment of water and carbon (Southwick 1984; Galen *et al.* 1999; Teixido and Valladares 2013). Consequently, the fitness costs of both traits should be higher in plants with little access to water and/or carbon compared to other plants. The result could be a positive relationship between petal size and nectar production in a plant population. (For other examples of resource deprivation having similar effects on both flower size and floral rewards, see Aizen and Raffaele 1996; Strauss *et al.* 1996; Burkle and Irwin 2009; see also Borghi *et al.* 2019).

Fitness benefits of floral signals and rewards might also be correlated with each other because both can contribute to the amount of pollen imported and exported from a plants’ flowers (as discussed in an earlier section). If a trait such as sexual morph or poor access to resources caused some plants to benefit less than others from pollen delivery and export, then the optimal investment in both signals and rewards might be lower in these ‘low-benefit’ plants compared to ‘high-benefit’ plants.

One caveat to this argument is that an increase in floral rewards can, in some cases, reduce a plant’s fitness. Increasing floral rewards typically increases the number of flower visits per plant visit (Brandenburg and Bshary 2011; Dreisig 2012), which can increase the rate of geitonogamy. In populations with high costs of geitonogamy, the optimal strategy for ‘high-benefit’ plants might therefore be to increase investment in signal attractiveness without a corresponding increase in floral rewards (e.g. Ishii and Harder 2006; Jersáková and Johnson 2006).

### Some promising areas for future research

The mixed results of studies assessing informative floral signals’ effects on plant attractiveness are intriguing and suggest a fruitful area for further research. Considerable previous work on the sensory abilities of pollinators (e.g. Chittka and Spaethe 2007; Wester and Lunau 2017), could provide a rich source of hypotheses about which types of signal are more or less likely to influence plant choice decisions, as well as how variables such as flower size and spacing between plants could affect how discriminating pollinators can be in their plant choices.

Even less is known about how informative signals influence pollinators’ probability of returning to a plant. Because the only study examining this relationship was carried out in a laboratory environment that necessarily constrained bees to forage at far fewer locations than they would under natural conditions, field research is needed to determine whether and under what conditions this behaviour is exhibited in the wild.

My verbal argument and some theoretical papers (e.g. Cohen and Shmida 1993; Sun *et al.* 2018) suggest variation across plants in costs and/or benefits of signals and rewards can stabilize an informative floral signal, provided that it is costly (Fig. 5B). However, we do not know whether those conditions are actually met by species with informative floral signals. Fitness costs of investing resources in signalling and producing rewards are difficult to measure (although see Pyke 1991), but fitness benefits of presenting attractive signals and floral rewards (as well as costs imposed by eavesdroppers) could be measured by manipulating those traits directly (e.g. Jersáková and Johnson 2006; Jersáková *et al.* 2008; Zhang *et al.* 2017). A first step, therefore, could be to determine whether the more-rewarding category of plants receives greater fitness benefits from both increased rewards and attractive signals than the less-rewarding category.

## Effects of informative signals on flower constancy and switching between sexes

### Within-plant reward information and flower constancy

Increased foraging success can increase pollinators’ flower constancy, that is, their probability of remaining constant to the same plant species while foraging (Chittka *et al.* 1997; Ishii 2005; Grüter *et al.* 2011). Wright and Schiestl (2009) proposed that floral signals that provided information about within-plant variation in floral rewards could increase that plant’s male fitness by encouraging the pollinator to remain flower constant when selecting its next plant (Fig. 4B). However, I am not aware of any study testing this idea.

### Effects of informative floral signals on frequency of switches between flower sexes

In plant species with separate sexual morphs, such as dioecious and gynodioecious species, both sexes can benefit from pollinators’ switching between sexes. A likely result of these benefits is selection on both sexes to resemble one another. However, if plants rewarded pollinators for moving from staminate to pistillate flowers, that might favour the evolution of signals that helped pollinators discriminate between sexes (Fig. 5C).

One way to reward movement between sexes could be to offer complementary resources in the two sexual morphs. Several beetle-pollinated species may exhibit this strategy (Hemborg and Bond 2005). For example, in *Leucadendron xanthoconus*, staminate flowers provide food as well as mating and egg-laying sites for their beetle pollinators, while pistillate flowers appear to provide shelter from frequent rains, encouraging trips between the sexes. These intersexual movements may be facilitated by the differences in inflorescence colour, size, shape and volatile emissions between the sexes. However, behavioural data are still needed to confirm this idea.

Another way to reward pollinators for moving from staminate to pistillate flowers is to make the reward contingent on pollination. Some brood-site pollination systems exhibit this strategy. For instance, many plant species in the tribe Phyllantheae are pollinated by *Epicephala* moths that actively collect pollen from staminate flowers and deposit it on pistillate flowers before laying their eggs in the flowers (Okamoto *et al.* 2013). The larvae feed on developing seeds and therefore rely on the flowers being pollinated. Circumstantial evidence that differences in floral signals promote pollination in this system comes from a survey of 11 species in the Phyllantheae, including representatives of several independent transitions to *Epicephala* moth pollination (Okamoto *et al.* 2013). Staminate and pistillate flowers in the seven species pollinated by *Epicephala* moths had qualitatively different scents, whereas the four species pollinated by other insects lacked this sexual dimorphism in scent.

## Conclusion

Although selective pressures on floral traits have captured biologists’ interest since the time of Darwin, the evolution of relationships between floral rewards and signals within plant species remains largely unexplored. Abundant evidence now shows that a variety of floral signals can provide information to pollinators about variation in floral rewards, both within and between plants, and that signal–reward correlations have arisen many times across widely dispersed plant taxa. In some cases, the rewards themselves are detectable before a pollinator lands on a flower (Fig. 1). In many other cases, floral signals are associated with characteristics such as flower age, pollination status, sexual phase or sexual morph that in turn are associated with floral reward production.

The effects of informative signals on pollinator behaviour, particularly decisions about whether to visit a plant, when to leave the plant and where to go next are poorly understood. Fitness effects of within-plant correlations between floral signals and rewards have rarely been measured. Where less-rewarding plants present different floral signals from more-rewarding plants, the reason why they fail to mimic more-rewarding plants is seldom known.

In this paper, I have discussed a variety of hypotheses to explain the evolution and maintenance of informative floral signals, focusing particularly on pollinator-mediated selective pressures (Figs 4 and 5). These hypotheses generate a variety of testable predictions, and I have suggested empirical approaches to testing many of them.

Given how common and widespread informative floral signals appear to be, we are surprisingly ignorant about the fitness effects and evolution of floral signal–reward correlations. My hope is that this paper will inspire readers to fill this substantial gap in our understanding of floral signals.

## Data Availability

This is a review article, and does not present any original data.

## Notes

**Populations & Communities. Chief Editor:** Jean Burns
